# Real-world safety profile of givinostat: an early post-marketing pharmacovigilance study based on the FAERS database

**DOI:** 10.3389/fphar.2026.1861893

**Published:** 2026-07-09

**Authors:** Jiahao Xu, Changhao Xiao, Liang Li, Xiaohui Ma, Shuang Xia, Hongjian Ji, Fengchao Shi

**Affiliations:** 1 Department of Orthopedics, Affiliated Hospital 6 of Nantong University, Yancheng, China; 2 Yancheng City Therapeutic Drug Precision Testing Engineering Center, School of Pharmacy, Jiangsu Medical College, Yancheng, China

**Keywords:** adverse events, disproportionality analysis, duchenne muscular dystrophy, FAERS, givinostat, pharmacovigilance

## Abstract

**Objective:**

Givinostat is a novel histone deacetylase inhibitor, which was approved by the US Food and Drug Administration (FDA) in 2024 for treatment of Duchenne muscular dystrophy (DMD). This early post-marketing pharmacovigilance study aimed to explore the adverse events (AEs) caused by givinostat through data mining of the US FDA Adverse Event Reporting System (FAERS), and provide reference for clinical safety.

**Methods:**

Data of givinostat were collected from the FAERS database covering the period from the first quarter of 2024 to the fourth quarter of 2025. Disproportionality analyses were employed to quantify the associated AE signals of givinostat and detect the risk signals from the data in the FAERS database. Four algorithms, including reporting odds ratio (ROR), proportional reporting ratio (PRR), Bayesian confidence propagation neural network (BCPNN), and empirical Bayes geometric mean (EBGM), were used to detect the risk signals from the data in the FAERS database. The definition relied on system organ class (SOCs) and preferred terms (PTs) by the Medical Dictionary for Regulatory Activities (MedDRA).

**Results:**

In total, 402 reports of givinostat were identified as the “primary suspected (PS)” AEs. Givinostat-associated AEs were identified across 23 organ systems. Significant disproportionality PTs simultaneously meeting the criteria of all four algorithms. Expected AEs, including decreased platelet count, diarrhoea, and increased blood triglycerides, were identified. Unexpected significant signals such as lower limb fracture, femur fracture, anger, and mood swings were also detected, although these are likely heavily confounded by the underlying disease progression and concurrent corticosteroid use.

**Conclusion:**

Based on early post-marketing data, this study provides a preliminary real-world safety assessment of givinostat. In addition to validating known hematological and gastrointestinal toxicities, it identifies potential signals for orthopedic complications, psychiatric events, and hypertriglyceridemia. While these novel signals are heavily confounded by the natural history of DMD and chronic corticosteroid exposure, they warrant continuous multidisciplinary monitoring. As hypothesis-generating signals derived from spontaneous reports, these initial findings require future prospective validation. They expand the current understanding of givinostat’s safety profile and highlight the necessity for continuous pharmacovigilance.

## 1 Introduction

Duchenne muscular dystrophy (DMD) is a severe X-linked recessive neuromuscular disorder primarily attributed to pathogenic variants within the DMD gene. The deficiency of functional dystrophin compromises the structural integrity of muscle fibers, rendering them highly susceptible to mechanical injury during repeated cycles of contraction. Consequently, the compromised muscle parenchyma undergoes progressive substitution by fibrotic and adipose tissue, accompanied by chronic, unrelenting inflammation throughout the clinical progression. Clinically, patients experience continuous loss of motor function, and many lose ambulation in childhood before later developing respiratory or cardiac failure (Duan et al., 2021).

Currently, the therapeutic landscape for DMD is evolving rapidly. While gene-targeted therapies, such as exon-skipping agents (e.g., eteplirsen, golodirsen) and adeno-associated virus (AAV)-mediated micro-dystrophin gene transfer, have brought groundbreaking hope, these interventions are highly mutation-specific and only applicable to a limited subset of the DMD population (Markati et al., 2022). At present, for the majority of patients, glucocorticoids are still conventional drugs in clinical practice. However, their clinical efficacy is still limited, and long-term use of glucocorticoids usually causes problems such as excessive weight gain, osteoporosis, fractures, and immunosuppression (McDonald et al., 2018). Therefore, there is still an urgent need for a new treatment method that is not limited by specific gene mutation subtypes.

In recent years, increasing attention has been paid to pathways related to histone deacetylase (HDAC) activity. Excessive HDAC signaling seems to aggravate regeneration impairment in dystrophic muscle and promote fibro-fatty remodeling. Givinostat is an oral HDAC inhibitor developed based on this rationale. It is reported that the drug can reduce the inflammatory response and affect the biological behavior of fibro-adipogenic progenitors involved in scar formation and adipogenesis (Aartsma-Rus, 2024). In March 2024, the US Food and Drug Administration (FDA) officially approved givinostat for the treatment of DMD patients aged 6 years and older (Lamb, 2024). Unlike mutation-specific therapies, its clinical application is not limited by specific genetic subtypes.

Recent clinical trials have demonstrated the clinical efficacy of givinostat (Bettica et al., 2016). In the Phase 3 EPIDYS trial, (Mercuri et al., 2024) found that boys treated with givinostat showed a slower decline in stair-climbing ability over 72 weeks than those in the placebo group. Imaging findings also pointed to reduced fat infiltration in the thigh muscles. Das et al. (2025), in a recent review, reached consistent conclusions. However, adverse events were common in the available studies, especially gastrointestinal reactions, thrombocytopenia, and hypertriglyceridemia, which sometimes necessitated dose reductions or temporary treatment interruptions.

That said, trial evidence does not necessarily reflect what happens after a drug enters routine practice. Rare events, delayed reactions, and toxicities in broader patient populations may be missed before approval. This is particularly relevant for a newly marketed treatment intended for children. Since givinostat was approved only recently, information from post-marketing surveillance remains very limited. FAERS is a large spontaneous reporting database frequently used to identify potential safety signals in the real world (Anand et al., 2019; Chen and Liang, 2026; Chen et al., 2026; Gao et al., 2025; He et al., 2025; Joshi et al., 2025; Le et al., 2026; Li et al., 2025; Lin et al., 2025a; b; Lu et al., 2026; Qu et al., 2024; Yin et al., 2022; Zhou et al., 2024; Zhu et al., 2025). In the present study, we used FAERS to examine early adverse event reports related to givinostat and to provide an initial description of its post-marketing safety profile.

## 2 Materials and methods

### 2.1 Study design and data source

Data for this study were retrieved from the FAERS database, available at:https://fis.fda.gov/extensions/FPD-QDE-FAERS/FPD-QDE-FAERS.html. All AE reports from the first quarter of 2024 to the fourth quarter of 2025 were systematically extracted. The downloaded raw ASCII data files were imported into the R 4.4.3 software environment for centralized data management and analysis. The extracted dataset comprehensively encompassed patient demographic information (DEMO), drug records (DRUG), patient outcomes (OUTC), AE details (REAC), report sources (RPSR), and therapy dates (THER).

### 2.2 Procedures

To identify givinostat-related AE reports, the generic name (“GIVINOSTAT”), trade name (“DUVYZAT”), and investigational compound codes (“ITF2357”and “ITF-2357”) were utilized to query the database. Data deduplication was strictly performed in accordance with FDA recommendations: for multiple records sharing the same Primary ID, older versions were discarded, and only the single case with the most recent reporting date or the highest version number was retained, thereby eliminating the risk of bias introduced by redundant data. During the subsequent screening phase, the dataset was restricted exclusively to reports where givinostat was designated as the “Primary Suspect” (PS) drug, aiming to control for potential confounding effects arising from polypharmacy. All extracted clinical AE terms were standardized and mapped using the Medical Dictionary for Regulatory Activities (MedDRA, v27.0). These terms were systematically classified at both the system organ class (SOC) and preferred term (PT) levels, establishing a standardized data foundation for subsequent signal mining. Prior to signal quantification, a prespecified data curation procedure was applied. Specifically, non-clinical adverse event terms, including the primary indication (e.g., “Duchenne Muscular Dystrophy”) and medication-error/product-quality terms (e.g., “Product Dose Omission Issue”), were systematically excluded from the final PT signal tables to ensure the analysis strictly focused on genuine drug-induced clinical safety signals.

### 2.3 Disproportionality analysis

For data mining and signal detection, this study employed the internationally recognized disproportionality analysis methodology. To robustly minimize the false-positive rate inherent to reliance on a single algorithm, a complementary approach combining four mainstream statistical algorithms was utilized. These included the frequentist methods—the reporting odds ratio (ROR) and proportional reporting ratio (PRR)—as well as the Bayesian methods—the Bayesian confidence propagation neural network (BCPNN) and the empirical Bayes geometric mean (EBGM). This multi-algorithm approach is widely recognized as a robust strategy to enhance signal reliability in recent pharmacovigilance research (Ye et al., 2025). The disproportionality analysis was conducted based on a classic 2 × 2 contingency table (Table 1). The values and corresponding 95% confidence intervals (CIs) for ROR, PRR, the Information Component (IC) of BCPNN, and EBGM were rigorously calculated according to established mathematical formulas and specific thresholds (Table 2). A higher calculated value indicates a stronger statistical association between givinostat exposure and a specific AE, suggesting that the AE represents a potential drug safety signal. Furthermore, to evaluate the robustness of our findings and address potential false-positive signals driven by extremely small sample sizes, a sensitivity analysis was conducted by restricting the data to AE signals with at least 4 reported cases (N ≥ 4).

**TABLE 1:** Four grid table.

Drug exposure	Target AEs	Non-target AEs	Total
Givinostat	a	b	a + b
Non-Givinostat	c	d	c + d
Total	a + c	b + d	N = a + b + c + d

**TABLE 2:** ROR, PRR, BCPNN, and EBGM methods, formulas, and thresholds.

Method	Formula	Threshold
ROR	$ROR=\frac{\left(a/c\right)}{\left(b/d\right)}=\frac{ad}{bc}$ $SE\left(\ln ROR\right)=\sqrt{\left(\frac{1}{a}+\frac{1}{b}+\frac{1}{c}+\frac{1}{d}\right)}95\%CI=e^{\ln\left(ROR\right)\pm 1.96\sqrt{\left(\frac{1}{a}+\frac{1}{b}+\frac{1}{c}+\frac{1}{d}\right)}}$	a ≥3 and 95% CI (lower limit) > 1
PRR	$PRR=\frac{a/\left(a+b\right)}{c/\left(c+d\right)}$ $SE\left(\ln PRR\right)=\sqrt{\frac{1}{a}-\frac{1}{a+b}+\frac{1}{c}-\frac{1}{c+d}}95\%CI=e^{\ln\left(PRR\right)\pm 1.96\sqrt{\frac{1}{a}-\frac{1}{a+b}+\frac{1}{c}-\frac{1}{c+d}}}$	a ≥3 and 95% CI (lower limit) > 1
BCPNN	$IC=\log_{2}\frac{p\left(x,y\right)}{p\left(x\right)p\left(y\right)}=\log_{2}\frac{a\left(a+b+c+d\right)}{\left(a+b\right)\left(a+c\right)}$ $E\left(IC\right)=\log_{2}\frac{\left(a+\gamma_{11}\right)\left(a+b+c+d+\alpha \right)\left(a+b+c+d+\beta \right)}{\left(a+b+c+d+\gamma \right)\left(a+b+\alpha_{1}\right)\left(a+c+\beta_{1}\right)}$ $V\left(IC\right)=\frac{1}{{\left(\ln2\right)}^{2}}\left\{\left[\frac{\left(a+b+c+d\right)-a+\gamma -\gamma_{11}}{\left(a+\gamma_{11}\right)\left(1+a+b+c+d+\gamma \right)}\right]+\left[\frac{\left(a+b+c+d\right)-\left(a+b\right)+\alpha -\alpha_{1}}{\left(a+b+\alpha_{1}\right)\left(1+a+b+c+d+\alpha \right)}\right]+\left[\frac{\left(a+b+c+d\right)-\left(a+c\right)+\beta -\beta_{1}}{\left(a+c+\beta_{1}\right)\left(1+a+b+c+d+\beta \right)}\right]\right\}$ $\gamma =\gamma_{11}\frac{\left(a+b+c+d+\alpha \right)\left(a+b+c+d+\beta \right)}{\left(a+b+\alpha_{1}\right)\left(a+c+\beta_{1}\right)}IC-2SD=E\left(IC\right)-2\sqrt{V\left(IC\right)}$	IC025 > 0
EBGM	$EBGM=\frac{a\left(a+b+c+d\right)}{\left(a+c\right)\left(a+b\right)}95\%CI=e^{\ln\left(EBGM\right)\pm 1.96\sqrt{\left(\frac{1}{a}+\frac{1}{b}+\frac{1}{c}+\frac{1}{d}\right)}}$	EBGM05 > 2

## 3 Results

### 3.1 General characteristics

**FIGURE 1:**
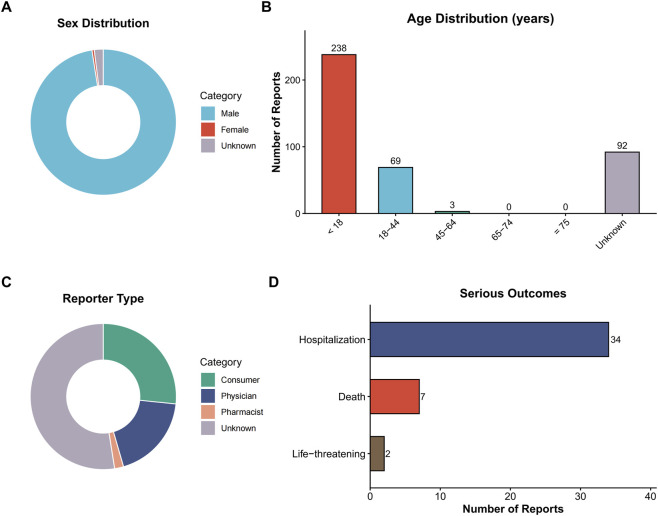
General characteristics of givinostat-associated adverse event reports in the FAERS database **(A)** Sex distribution of the patients **(B)** Age distribution stratified by groups **(C)** Proportion of different reporter types **(D)** Frequencies of serious clinical outcomes resulting from the adverse events.

The clinical characteristics of givinostat-associated AEs are described in Table 3. For gender, the incidence of AEs in males (97.51%) accounted for a larger proportion than females (0.50%). In terms of age composition, patients aged under 18 years accounted for a higher proportion (59.20%) than patients aged between 18 and 44 years old (17.16%). The United States (96.27%) reported the largest number of AEs, followed by the United Kingdom (1.74%), Germany (1.00%), and France (0.50%). Serious outcomes included death, life-threatening, and hospitalization. Hospitalization was the most frequently reported serious outcome in 34 (8.46%) cases. Other serious outcomes including death and life-threatening were reported in 7 (1.74%) and 2 (0.50%) cases, respectively. Excluding the unknown reporters, consumers and physicians reported the most AEs for 26.62% and 18.91%, respectively. The number of AEs reported were submitted in 2025 (94.28%), followed by 2024 (5.72%). The detailed demographic and clinical characteristics of these 402 adverse event reports are illustrated in Figure 1, including gender distribution (Figure 1A), age grouping (Figure 1B), reporter type composition (Figure 1C), and the frequency of serious clinical outcomes (Figure 1D).

**TABLE 3:** Clinical characteristics of reports associated with givinostat.

Characteristics	Number of cases	Proportion of cases (%)
Number of adverse events	402	​
Year of report		
2024	23	5.72
2025	379	94.28
Sex		
Female	2	0.50
Male	392	97.51
Unknown	8	1.99
Age (years)		
<18	238	59.20
18–44	69	17.16
45–64	3	0.75
65–74	0	0.00
≥75	0	0.00
Unknown	92	22.89
Reported countries		
United States	387	96.27
United Kingdom	7	1.74
Germany	4	1.00
France	2	0.50
European union	2	0.50
Serious outcome		
Death	7	1.74
Life-threatening	2	0.50
Hospitalization	34	8.46
Disability	0	0.00
Reporter		
Consumer	107	26.62
Physician	76	18.91
Pharmacist	8	1.99
Other health professionals	0	0.00
Lawyer	0	0.00
Unknown	211	52.49

### 3.2 Signal detection

**FIGURE 2:**
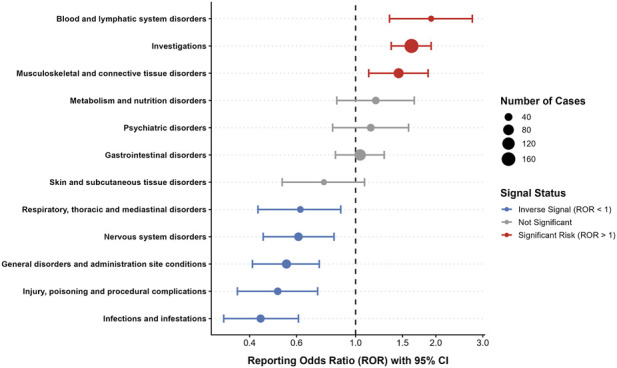
Forest plot of Reporting Odds Ratios (RORs) for givinostat adverse events by System Organ Class (SOC). Red denotes significant risk signals (lower 95% CI > 1), blue indicates inverse signals (upper 95% CI < 1), and grey signifies non-significant associations. Dot size corresponds to the log10 of case counts. The vertical dashed line indicates ROR = 1.

The signal strengths of givinostat-associated AEs at the System Organ Class (SOC) level are described in Table 4 and visually presented in Figure 2. Our analysis revealed that 23 organ systems were involved in givinostat-associated AEs. A positive safety signal was defined as meeting all four algorithm criteria simultaneously: reporting odds ratio (ROR) of ≥3 and 95% confidence interval (CI) lower bound >1; proportional reporting ratio (PRR) with a ≥3 and 95% CI lower bound >1; Bayesian confidence propagation neural network (BCPNN) with IC025 > 0; and empirical Bayes geometric mean (EBGM) with EBGM05 > 2. Based on the data, the most prominent significant risk signals (lower limit of 95% CI > 1) were investigations, gastrointestinal disorders, and blood and lymphatic system disorders. Notably, investigations was the only SOC that strictly met all four criteria. Gastrointestinal disorders and blood and lymphatic system disorders were significant SOCs where at least one of the four indices met the criteria. Conversely, several SOCs, including injury, poisoning and procedural complications, general disorders and administration site conditions, and infections and infestations, presented as inverse signals (upper limit of 95% CI < 1), reflecting relatively lower reporting frequencies.

**TABLE 4:** The signal strength of AEs of Givinostat at the SOC level.

System organ class	SOC code	Case reports	ROR (95% CI)	PRR (95% CI)	χ2	IC (IC025)	EBGM (EBGM05)
Investigations	10,022,891	172	5.84 (4.80–7.12)	3.77 (3.37–4.22)	394.82	1.90 (1.69)	3.77 (3.25)
Gastrointestinal disorders	10,017,947	141	3.01 (2.45–3.69)	2.30 (2.02–2.63)	122.59	1.20 (0.96)	2.30 (1.95)
Injury, poisoning and procedural complications	10,022,117	77	0.51 (0.40–0.65)	0.60 (0.49–0.74)	29.81	−0.73 (−1.05)	0.60 (0.48)
General disorders and administration site conditions	10,018,065	70	0.36 (0.27–0.46)	0.47 (0.38–0.58)	67.71	−1.09 (−1.43)	0.47 (0.37)
Infections and infestations	1,0,021,881	37	0.70 (0.50–0.99)	0.73 (0.54–0.99)	4.21	−0.45 (−0.91)	0.73 (0.53)
Blood and lymphatic system disorders	10,005,329	33	1.92 (1.35–2.75)	1.85 (1.33–2.56)	13.42	0.87 (0.38)	1.85 (1.31)
Nervous system disorders	10,029,205	30	0.45 (0.31–0.65)	0.49 (0.35–0.69)	18.83	−1.02 (−1.53)	0.49 (0.34)
Skin and subcutaneous tissue disorders	10,040,785	29	0.58 (0.40–0.85)	0.61 (0.43–0.87)	8.02	−0.70 (−1.22)	0.61 (0.43)
Musculoskeletal and connective tissue disorders	10,028,395	26	0.68 (0.45–1.01)	0.70 (0.48–1.01)	3.77	−0.51 (−1.06)	0.70 (0.47)
Psychiatric disorders	10,037,175	20	0.56 (0.36–0.87)	0.58 (0.38–0.89)	6.66	−0.77 (−1.40)	0.58 (0.37)
Respiratory, thoracic and mediastinal disorders	10,038,738	20	0.49 (0.31–0.77)	0.52 (0.34–0.79)	9.89	−0.93 (−1.55)	0.52 (0.33)
Metabolism and nutrition disorders	10,027,433	16	0.76 (0.46–1.26)	0.77 (0.48–1.25)	1.15	−0.37 (−1.06)	0.77 (0.47)
Cardiac disorders	10,007,541	10	0.53 (0.28–0.99)	0.54 (0.29–1.00)	4.11	−0.86 (−1.73)	0.54 (0.29)
Vascular disorders	10,047,065	7	0.33 (0.15–0.69)	0.34 (0.16–0.70)	9.63	−1.50 (−2.54)	0.34 (0.16)
Immune system disorders	10,021,428	6	0.42 (0.19–0.94)	0.43 (0.19–0.95)	4.75	−1.16 (−2.27)	0.43 (0.19)
Renal and urinary disorders	10,038,359	6	0.38 (0.17–0.84)	0.39 (0.17–0.85)	6.12	−1.31 (−2.42)	0.39 (0.17)
Surgical and medical procedures	10,042,613	6	0.29 (0.13–0.65)	0.30 (0.14–0.67)	10.23	−1.65 (−2.76)	0.30 (0.14)
Congenital, familial and genetic disorders	10,010,331	4	1.68 (0.63–4.51)	1.68 (0.63–4.45)	1.10	0.64 (−0.69)	1.68 (0.63)
Ear and labyrinth disorders	10,013,993	4	0.89 (0.33–2.37)	0.89 (0.33–2.35)	0.06	−0.15 (−1.49)	0.89 (0.33)
Social circumstances	10,041,244	4	0.66 (0.25–1.77)	0.66 (0.25–1.76)	0.69	−0.54 (−1.87)	0.66 (0.25)
Product issues	10,077,536	3	0.12 (0.04–0.38)	0.13 (0.04–0.40)	18.62	−2.76 (−4.27)	0.13 (0.04)
Endocrine disorders	10,014,698	1	0.29 (0.04–2.08)	0.29 (0.04–2.09)	1.70	−1.38 (−3.68)	0.29 (0.04)
Hepatobiliary disorders	10,019,805	1	0.10 (0.01–0.69)	0.10 (0.01–0.70)	8.37	−2.82 (−5.12)	0.10 (0.01)

**FIGURE 3:**
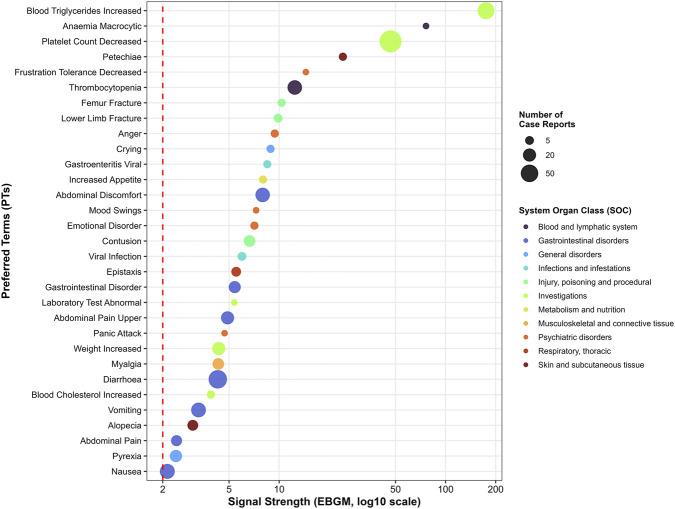
Bubble chart of key significant adverse event (AE) signals associated with givinostat at the Preferred Term (PT) level. The y-axis displays the specific PTs, while the x-axis represents the signal strength measured by the Empirical Bayes Geometric Mean (EBGM) on a logarithmic (log10) scale. The size of the bubbles is strictly proportional to the number of case reports for each respective AE. The colors of the bubbles denote the corresponding System Organ Class (SOC) categories. The vertical red dashed line indicates the statistical significance threshold of EBGM = 2.0.

In accordance with our prespecified curation procedure, non-adverse event terms such as the primary indication and medication errors were excluded. The curated clinical PTs with significant disproportionality conforming to the four algorithms are shown in Tables 5, 6, and their signal strengths are visually summarized in Figure 3. To confirm the stability of these signals, a sensitivity analysis retaining only PTs with ≥4 cases was performed. The core safety signals remained highly robust, as detailed in Supplementary Table S1. Additionally, exploratory signals excluded from the primary analysis due to low report counts (N ≤ 3) are provided in Supplementary Table S2. According to previous studies of givinostat, hematological abnormalities and gastrointestinal issues are usually reported. In our study, platelet count decreased (n = 97), diarrhoea (n = 59), blood triglycerides increased (n = 45), nausea (n = 31), vomiting (n = 30), and thrombocytopenia (n = 28) were consistent with findings from clinical trials. Interestingly, significant disproportionality signals not listed in the label were discovered, including lower limb fracture (EBGM: 9.86), femur fracture (EBGM: 10.35), frustration tolerance decreased (EBGM: 14.47), anger (EBGM: 9.41), and mood swings (EBGM: 7.27). However, these signals must be interpreted with extreme caution due to the strong confounding effects of the underlying disease and co-medications.

**TABLE 5:** The top positive AEs of givinostat ranked by case reports at the PT level.

SOC	PTs	Case reports	ROR (95% CI)	PRR (95% CI)	χ2	IC (IC025)	EBGM (EBGM05)
Investigations	Platelet count decreased	97	61.58 (48.98–77.43)	46.96 (39.46–55.90)	4,357.25	5.24 (4.95)	46.66 (38.24)
Gastrointestinal disorders	Diarrhoea	59	4.85 (3.68–6.40)	4.29 (3.39–5.43)	153.84	2.06 (1.69)	4.28 (3.32)
Investigations	Blood triglycerides increased	45	201.97 (147.61–276.34)	179.47 (135.74–237.29)	7,792.96	5.91 (5.49)	175.04 (130.69)
Gastrointestinal disorders	Nausea	31	2.23 (1.54–3.21)	2.13 (1.52–2.99)	19.32	1.07 (0.56)	2.13 (1.50)
Gastrointestinal disorders	Vomiting	30	3.46 (2.39–5.02)	3.28 (2.32–4.63)	48.62	1.66 (1.15)	3.28 (2.29)
Gastrointestinal disorders	Abdominal discomfort	28	8.49 (5.78–12.47)	7.97 (5.57–11.39)	171.97	2.83 (2.30)	7.96 (5.50)
Blood and lymphatic system disorders	Thrombocytopenia	28	13.28 (9.04–19.51)	12.43 (8.69–17.77)	295.32	3.37 (2.84)	12.41 (8.57)
Gastrointestinal disorders	Abdominal pain upper	20	5.11 (3.26–8.01)	4.90 (3.20–7.52)	62.71	2.16 (1.54)	4.90 (3.16)
Investigations	Weight increased	20	4.51 (2.88–7.08)	4.34 (2.83–6.65)	51.96	2.00 (1.38)	4.34 (2.80)
General disorders and administration site conditions	Pyrexia	16	2.46 (1.49–4.06)	2.40 (1.49–3.88)	13.30	1.20 (0.51)	2.40 (1.47)
Gastrointestinal disorders	Gastrointestinal disorder	15	5.59 (3.34–9.36)	5.42 (3.30–8.90)	54.37	2.24 (1.53)	5.41 (3.26)
Injury, poisoning and procedural complications	Contusion	13	6.84 (3.93–11.89)	6.65 (3.89–11.35)	62.65	2.46 (1.69)	6.64 (3.86)
Musculoskeletal and connective tissue disorders	Myalgia	13	4.43 (2.55–7.69)	4.31 (2.53–7.37)	33.33	1.94 (1.17)	4.31 (2.50)
Gastrointestinal disorders	Abdominal pain	11	2.46 (1.35–4.48)	2.42 (1.35–4.34)	9.29	1.19 (0.36)	2.42 (1.34)
Skin and subcutaneous tissue disorders	Alopecia	10	3.08 (1.64–5.77)	3.03 (1.64–5.58)	13.68	1.46 (0.59)	3.03 (1.63)
Respiratory, thoracic and mediastinal disorders	Epistaxis	7	5.60 (2.65–11.83)	5.52 (2.65–11.51)	25.97	2.08 (1.05)	5.52 (2.63)
Injury, poisoning and procedural complications	Lower limb fracture	5	9.98 (4.13–24.13)	9.87 (4.13–23.60)	39.85	2.45 (1.24)	9.86 (4.10)
Infections and infestations	Viral infection	5	6.05 (2.50–14.62)	5.99 (2.50–14.31)	20.79	2.04 (0.84)	5.98 (2.49)
Psychiatric disorders	Anger	4	9.51 (3.55–25.47)	9.42 (3.55–25.00)	30.11	2.28 (0.95)	9.41 (3.53)
Investigations	Blood cholesterol increased	4	3.92 (1.46–10.50)	3.89 (1.47–10.32)	8.61	1.56 (0.23)	3.89 (1.46)
General disorders and administration site conditions	Crying	4	8.97 (3.35–24.03)	8.89 (3.35–23.59)	28.01	2.24 (0.91)	8.88 (3.33)
Psychiatric disorders	Emotional disorder	4	7.16 (2.67–19.19)	7.10 (2.68–18.84)	20.99	2.08 (0.75)	7.10 (2.66)
Injury, poisoning and procedural complications	Femur fracture	4	10.46 (3.90–28.03)	10.37 (3.91–27.50)	33.83	2.34 (1.01)	10.35 (3.89)
Infections and infestations	Gastroenteritis viral	4	8.57 (3.20–22.96)	8.50 (3.20–22.54)	26.45	2.21 (0.88)	8.49 (3.19)
Metabolism and nutrition disorders	Increased appetite	4	8.07 (3.01–21.63)	8.00 (3.02–21.23)	24.52	2.17 (0.84)	8.00 (3.00)
Skin and subcutaneous tissue disorders	Petechiae	4	24.46 (9.12–65.60)	24.22 (9.12–64.34)	88.79	2.76 (1.42)	24.14 (9.06)
Blood and lymphatic system disorders	Anaemia macrocytic	3	77.69 (24.80–243.40)	77.12 (24.82–239.58)	222.97	2.70 (1.19)	76.29 (24.61)
Psychiatric disorders	Frustration tolerance decreased	3	14.60 (4.68–45.53)	14.50 (4.69–44.83)	37.66	2.31 (0.80)	14.47 (4.67)
Investigations	Laboratory test abnormal	3	5.42 (1.74–16.87)	5.38 (1.74–16.63)	10.71	1.73 (0.22)	5.38 (1.74)
Psychiatric disorders	Mood swings	3	7.32 (2.35–22.82)	7.28 (2.36–22.48)	16.24	1.94 (0.43)	7.27 (2.34)
Psychiatric disorders	Panic attack	3	4.73 (1.52–14.73)	4.70 (1.52–14.52)	8.75	1.62 (0.11)	4.70 (1.52)

**TABLE 6:** The top signal strength of AEs of givinostat ranked by EBGM at the PTs level.

SOC	PTs	Case reports	ROR (95% CI)	PRR (95% CI)	χ2	IC (IC025)	EBGM (EBGM05)
Investigations	Blood triglycerides increased	45	201.97 (147.61–276.34)	179.47 (135.74–237.29)	7,792.96	5.91 (5.49)	175.04 (130.69)
Blood and lymphatic system disorders	Anaemia macrocytic	3	77.69 (24.80–243.40)	77.12 (24.82–239.58)	222.97	2.70 (1.19)	76.29 (24.61)
Investigations	Platelet count decreased	97	61.58 (48.98–77.43)	46.96 (39.46–55.90)	4,357.25	5.24 (4.95)	46.66 (38.24)
Skin and subcutaneous tissue disorders	Petechiae	4	24.46 (9.12–65.60)	24.22 (9.12–64.34)	88.79	2.76 (1.42)	24.14 (9.06)
Psychiatric disorders	Frustration tolerance decreased	3	14.60 (4.68–45.53)	14.50 (4.69–44.83)	37.66	2.31 (0.80)	14.47 (4.67)
Blood and lymphatic system disorders	Thrombocytopenia	28	13.28 (9.04–19.51)	12.43 (8.69–17.77)	295.32	3.37 (2.84)	12.41 (8.57)
Injury, poisoning and procedural complications	Femur fracture	4	10.46 (3.90–28.03)	10.37 (3.91–27.50)	33.83	2.34 (1.01)	10.35 (3.89)
Injury, poisoning and procedural complications	Lower limb fracture	5	9.98 (4.13–24.13)	9.87 (4.13–23.60)	39.85	2.45 (1.24)	9.86 (4.10)
Psychiatric disorders	Anger	4	9.51 (3.55–25.47)	9.42 (3.55–25.00)	30.11	2.28 (0.95)	9.41 (3.53)
General disorders and administration site conditions	Crying	4	8.97 (3.35–24.03)	8.89 (3.35–23.59)	28.01	2.24 (0.91)	8.88 (3.33)
Infections and infestations	Gastroenteritis viral	4	8.57 (3.20–22.96)	8.50 (3.20–22.54)	26.45	2.21 (0.88)	8.49 (3.19)
Metabolism and nutrition disorders	Increased appetite	4	8.07 (3.01–21.63)	8.00 (3.02–21.23)	24.52	2.17 (0.84)	8.00 (3.00)
Gastrointestinal disorders	Abdominal discomfort	28	8.49 (5.78–12.47)	7.97 (5.57–11.39)	171.97	2.83 (2.30)	7.96 (5.50)
Psychiatric disorders	Mood swings	3	7.32 (2.35–22.82)	7.28 (2.36–22.48)	16.24	1.94 (0.43)	7.27 (2.34)
Psychiatric disorders	Emotional disorder	4	7.16 (2.67–19.19)	7.10 (2.68–18.84)	20.99	2.08 (0.75)	7.10 (2.66)
Injury, poisoning and procedural complications	Contusion	13	6.84 (3.93–11.89)	6.65 (3.89–11.35)	62.65	2.46 (1.69)	6.64 (3.86)
Infections and infestations	Viral infection	5	6.05 (2.50–14.62)	5.99 (2.50–14.31)	20.79	2.04 (0.84)	5.98 (2.49)
Respiratory, thoracic and mediastinal disorders	Epistaxis	7	5.60 (2.65–11.83)	5.52 (2.65–11.51)	25.97	2.08 (1.05)	5.52 (2.63)
Gastrointestinal disorders	Gastrointestinal disorder	15	5.59 (3.34–9.36)	5.42 (3.30–8.90)	54.37	2.24 (1.53)	5.41 (3.26)
Investigations	Laboratory test abnormal	3	5.42 (1.74–16.87)	5.38 (1.74–16.63)	10.71	1.73 (0.22)	5.38 (1.74)
Gastrointestinal disorders	Abdominal pain upper	20	5.11 (3.26–8.01)	4.90 (3.20–7.52)	62.71	2.16 (1.54)	4.90 (3.16)
Psychiatric disorders	Panic attack	3	4.73 (1.52–14.73)	4.70 (1.52–14.52)	8.75	1.62 (0.11)	4.70 (1.52)
Investigations	Weight increased	20	4.51 (2.88–7.08)	4.34 (2.83–6.65)	51.96	2.00 (1.38)	4.34 (2.80)
Musculoskeletal and connective tissue disorders	Myalgia	13	4.43 (2.55–7.69)	4.31 (2.53–7.37)	33.33	1.94 (1.17)	4.31 (2.50)
Gastrointestinal disorders	Diarrhoea	59	4.85 (3.68–6.40)	4.29 (3.39–5.43)	153.84	2.06 (1.69)	4.28 (3.32)
Investigations	Blood cholesterol increased	4	3.92 (1.46–10.50)	3.89 (1.47–10.32)	8.61	1.56 (0.23)	3.89 (1.46)
Gastrointestinal disorders	Vomiting	30	3.46 (2.39–5.02)	3.28 (2.32–4.63)	48.62	1.66 (1.15)	3.28 (2.29)
Skin and subcutaneous tissue disorders	Alopecia	10	3.08 (1.64–5.77)	3.03 (1.64–5.58)	13.68	1.46 (0.59)	3.03 (1.63)
Gastrointestinal disorders	Abdominal pain	11	2.46 (1.35–4.48)	2.42 (1.35–4.34)	9.29	1.19 (0.36)	2.42 (1.34)
General disorders and administration site conditions	Pyrexia	16	2.46 (1.49–4.06)	2.40 (1.49–3.88)	13.30	1.20 (0.51)	2.40 (1.47)
Gastrointestinal disorders	Nausea	31	2.23 (1.54–3.21)	2.13 (1.52–2.99)	19.32	1.07 (0.56)	2.13 (1.50)

## 4 Discussion

DMD is a severe X-linked recessive disorder caused by pathogenic variants in the DMD gene, resulting in dystrophin deficiency. This primary defect destabilizes the dystrophin-associated protein complex (DAPC), leading to extreme sarcolemmal fragility, dysregulated calcium homeostasis, and oxidative stress (Bez Batti Angulski et al., 2023; Chang et al., 2023). Notably, fast glycolytic muscle fibers exhibit significantly higher vulnerability to this contraction-induced mechanical stress compared to slow oxidative fibers, driving progressive myofiber degeneration (Bonato et al., 2024). This chronic damage is accompanied by massive epigenetic and metabolic alterations. For instance, specific microRNAs (miRNAs) are severely dysregulated, exacerbating NF-κB-mediated chronic inflammation, oxidative stress, and fibro-adipose replacement of muscle tissue (Aguilar et al., 2025). Furthermore, secondary downstream mechanisms, such as lipin1 deficiency, compromise membrane integrity and promote myofiber necroptosis (Jama et al., 2024).

Given this multifaceted pathology, the clinical management of DMD is highly complex. Standard-of-care therapy relies heavily on corticosteroids, which can delay loss of ambulation but are associated with substantial long-term toxicities, complicating the assessment of clinical outcome measures in clinical trials (Benemei et al., 2025). Consequently, diverse therapeutic strategies have been aggressively developed. Dystrophin-restoring modalities include exon-skipping and recent breakthroughs in AAV-mediated micro-dystrophin gene therapy, such as delandistrogene moxeparvovec (Mendell et al., 2025). In parallel, dystrophin-independent strategies aim to mitigate downstream pathological cascades—such as promoting a slow-oxidative muscle phenotype, restoring lipin1, or utilizing anti-inflammatory and anti-fibrotic small molecules (Bez Batti Angulski et al., 2023; Chang et al., 2023). This broad array of baseline pathophysiological factors and concomitant therapies inherently introduces significant disease-related confounding (disease bias) when evaluating the safety of novel agents.

Against this highly complex clinical backdrop, givinostat represents a newly marketed orphan drug for the treatment of DMD with limited real-world evidence, characterized by a relatively brief period of clinical application. The objective of this study is to furnish an early safety alert regarding this drug; consequently, medication errors such as “product dose omission issue” were excluded from the ensuing discussion.

In our disproportionality analysis, the SOC most reported by givinostat is mainly investigations and gastrointestinal disorders. In terms of PT, the most frequently reported are hematological and gastrointestinal events, such as platelet count decreased (ROR = 61.58), thrombocytopenia (ROR = 13.28) and diarrhoea (ROR = 4.85). These findings are highly consistent with the phase III clinical trials of the drug (Mercuri et al., 2024) and the adverse reactions mentioned in the FDA-approved drug label.

Givinostat is an HDAC inhibitor, and thrombocytopenia is a common adverse reaction of these drugs (Das et al., 2025; O'Connor et al., 2015; Rai et al., 2023). A preclinical study published in the journal Blood confirmed that HDAC inhibitors mainly hinder the maturation and differentiation of bone marrow megakaryocytes to reduce the production and release of platelets, rather than directly destroy platelets in the peripheral circulation (Bishton et al., 2011). Therefore, it is necessary to monitor the whole blood cell count (CBC) of patients during givinostat treatment.

Notably, our study detected exceptionally high disproportionality signals for lower limb fractures (ROR = 9.98) and femoral fractures (ROR = 10.46). However, we must strictly emphasize that these findings represent a classic statistical artifact driven by confounding by indication and co-medication, rather than a novel toxicological signal of givinostat. This methodological challenge of distinguishing true adverse reactions from disease-related confounding is a recognized phenomenon in real-world safety evaluations of orphan drugs for rare genetic disorders (Hong and Zhou, 2025). Because givinostat is exclusively prescribed to boys with DMD—a population where up to 20%–60% inherently experience low-trauma long bone fractures due to progressive myopathy and chronic corticosteroid exposure (Birnkrant et al., 2018; King et al., 2007)—the baseline fracture risk of the exposed cohort is fundamentally asymmetrical compared to the general FAERS population. Consequently, the elevated ROR values merely reflect the natural history of DMD and the accumulated toxicity of background glucocorticoids, rather than the direct osteotoxicity of givinostat. Given the inherent vulnerability of this specific patient population, multidisciplinary monitoring—including dynamic bone mineral density assessment (Pirih et al., 2012; Tian and Yu, 2015; Zhan et al., 2023) and fall prevention—remains essential during treatment.

By the same epidemiological logic, the strong signals detected for psychiatric adverse events—such as anger, mood swings, and panic attacks—are heavily confounded by the underlying disease. As demonstrated by Ricotti et al., 24% of boys with DMD exhibit clinically significant internalizing behaviors, and 15% display prominent externalizing behaviors at baseline, and over one-third present with two or more concurrent emotional or neurobehavioral comorbidities (Ricotti et al., 2016). The absence of dystrophin in the central nervous system inherently disrupts the structure and function of central inhibitory GABAergic synapses, making these neuropsychiatric symptoms part of the DMD clinical spectrum (Waite et al., 2012). Therefore, these psychiatric signals captured by our algorithms should be interpreted as the background noise of the treated population rather than adverse reactions caused by givinostat.

Apart from the confounded signals, it is worth noting that our study found a very significant signal for hypertriglycerides increased (ROR = 201.97). This is a genuine and critical metabolic adverse event associated with givinostat. Givinostat may induce abnormal lipid metabolism, which requires close clinical attention. Consequently, these findings generate the hypothesis that proactive lipid monitoring might be beneficial for patients receiving givinostat, which warrants further prospective clinical validation.

Furthermore, to provide a more granular clinical description of these genuine adverse events, we conducted a time-to-onset (TTO) analysis for the major signals (Supplementary Table S5). The median TTO for gastrointestinal disorders was highly acute at 2.0 days (IQR: 0.0–15.2), aligning with the direct mucosal irritation typical of oral medications. In contrast, hematological toxicity and hypertriglyceridemia exhibited a delayed onset pattern, with median TTOs of 19.0 days (IQR: 14.0–39.5) and 21.5 days (IQR: 14.0–52.0), respectively. This temporal divergence strongly suggests that while gastrointestinal monitoring should be intensified immediately upon treatment initiation, vigilance for hematological and metabolic shifts (such as triglyceride elevation) must be maintained progressively over the first month of therapy.

We observed an ROR below 1 in specific SOC categories, such as “injury, poisoning and procedural complications” (ROR = 0.51). This statistical phenomenon should not be misinterpreted as a protective effect of the drug. Rather, it highlights a typical underreporting bias in pharmacovigilance data. Because progressive muscle weakness makes DMD patients highly susceptible to accidental falls, clinicians usually view these events as natural disease progression. Therefore, they rarely report them to the FDA as drug-induced adverse events. Additionally, given the limited real-world exposure since the drug’s recent approval, the distribution of reported adverse events across different body systems remains somewhat imbalanced.

There are several inevitable limitations to this study design. Due to the recent market availability of givinostat and the rare nature of DMD, the accumulated sample size in the database remains relatively small (N = 402). Therefore, these early signals serve primarily as valuable clues for future research. We also face the common drawbacks of such databases, primarily inevitable underreporting. Conversely, the recent market entry of the drug might also trigger the “Weber effect,” temporarily inflating the reporting frequency of certain AEs (Hoffman et al., 2014). To descriptively assess this potential bias, we stratified the major signals by reporting year (Supplementary Table S3). Because only 23 reports were available in 2024, full year-stratified disproportionality analyses were underpowered. Nevertheless, the descriptive distribution confirms that key signals are stably concentrated in 2025 rather than the initial approval year, indicating they are not merely early reporting artifacts. Furthermore, as a valuable methodological consideration, performing a restricted sensitivity analysis using active comparators (such as deflazacort, eteplirsen, or vorinostat) would ideally mitigate confounding by indication. However, a rigorous feasibility assessment revealed that performing a full active-comparator analysis is currently impeded by significant data sparsity and methodological constraints. As detailed in Supplementary Table S4, querying the FAERS database for potential comparators—including DMD-indicated agents (e.g., deflazacort, eteplirsen, golodirsen), other HDAC inhibitors (e.g., vorinostat, romidepsin, panobinostat), and standard-of-care corticosteroids (prednisone)—resulted in critical limitations such as zero-cell problems in the 2 × 2 contingency tables, highly unstable confidence intervals, and massive background confounding. Consequently, reliable disproportionality signals could not be calculated using this approach at present, making our transparent reporting of these limitations essential. Another technical challenge is the absence of denominator data—without knowing the total number of global prescriptions, it is impossible to calculate absolute AE incidence rates (Michel et al., 2017). From a clinical perspective, FAERS reports rarely provide a complete patient history. Considering that nearly all boys with DMD are maintained on background corticosteroids, clearly distinguishing givinostat-induced adverse effects from the confounding effects of polypharmacy remains exceedingly difficult. To resolve these uncertainties, particularly regarding the drug’s long-term impact on bone microarchitecture and metabolism, the field will inevitably require extended real-world prospective cohorts.

## 5 Conclusion

In summary, this study provides the first real-world pharmacovigilance landscape of givinostat. Beyond expected side effects like thrombocytopenia and gastrointestinal toxicity, our analysis identified signals of orthopedic complications, hypertriglyceridemia, and psychiatric abnormalities. Crucially, the observed bone and psychiatric events likely reflect the cumulative toxicity of background corticosteroids and the natural progression of DMD, rather than direct drug toxicity. These findings highlight the necessity of a multidisciplinary team (MDT) approach. We propose that a multidisciplinary team (MDT) approach might be beneficial for patient management. Rather than defining standard clinical protocols, our findings should be strictly viewed as hypothesis-generating signals that highlight the potential value of lipid monitoring, periodic BMD follow-up, and fall prevention. Ultimately, these real-world insights require future prospective validation to definitively guide the safe application of givinostat.

## Data Availability

The original contributions presented in the study are included in the article/Supplementary Material, further inquiries can be directed to the corresponding authors.

## References

[B1] Aartsma-Rus A. (2024). Histone deacetylase inhibition with givinostat: a multi-targeted mode of action with the potential to halt the pathological cascade of Duchenne muscular dystrophy. Front. Cell Developmental Biology 12, 1514898. 10.3389/fcell.2024.1514898 39834392 PMC11743666

[B2] Aguilar J. Almeida-Becerril T. Rodríguez-Cruz M. (2025). Advances in MicroRNAs in pathophysiology of duchenne muscular dystrophy. Muscle and Nerve 72 (4), 541–555. 10.1002/mus.28482 40682311

[B3] Anand K. Ensor J. Trachtenberg B. Bernicker E. H. (2019). Osimertinib-induced cardiotoxicity: a retrospective review of the FDA adverse events reporting system (FAERS). JACC. CardioOncology 1 (2), 172–178. 10.1016/j.jaccao.2019.10.006 34396179 PMC8352117

[B4] Benemei S. Gatto F. Boni L. Pane M. (2025). If you cannot measure it, you cannot improve it. Outcome measures in Duchenne muscular dystrophy: current and future perspectives. Acta Neurol. Belg. 125 (1), 1–12. 10.1007/s13760-024-02600-2 39080230 PMC11876273

[B5] Bettica P. Petrini S. D'Oria V. D'Amico A. Catteruccia M. Pane M. (2016). Histological effects of givinostat in boys with Duchenne muscular dystrophy. Neuromuscul. Disorders NMD 26 (10), 643–649. 10.1016/j.nmd.2016.07.002 27566866

[B6] Bez Batti Angulski A. Hosny N. Cohen H. Martin A. A. Hahn D. Bauer J. (2023). Duchenne muscular dystrophy: disease mechanism and therapeutic strategies. Front. Physiology 14, 1183101. 10.3389/fphys.2023.1183101 37435300 PMC10330733

[B7] Birnkrant D. J. Bushby K. Bann C. M. Alman B. A. Apkon S. D. Blackwell A. (2018). Diagnosis and management of Duchenne muscular dystrophy, part 2: respiratory, cardiac, bone health, and orthopaedic management. Lancet. Neurology 17 (4), 347–361. 10.1016/S1474-4422(18)30025-5 29395990 PMC5889091

[B8] Bishton M. J. Harrison S. J. Martin B. P. McLaughlin N. James C. Josefsson E. C. (2011). Deciphering the molecular and biologic processes that mediate histone deacetylase inhibitor-induced thrombocytopenia. Blood 117 (13), 3658–3668. 10.1182/blood-2010-11-318055 21292776

[B9] Bonato A. Raparelli G. Caruso M. (2024). Molecular pathways involved in the control of contractile and metabolic properties of skeletal muscle fibers as potential therapeutic targets for Duchenne muscular dystrophy. Front. Physiology 15, 1496870. 10.3389/fphys.2024.1496870 39717824 PMC11663947

[B10] Chang M. Cai Y. Gao Z. Chen X. Liu B. Zhang C. (2023). Duchenne muscular dystrophy: pathogenesis and promising therapies. J. Neurology 270 (8), 3733–3749. 10.1007/s00415-023-11796-x 37258941

[B11] Chen T. Liang M. (2026). Real-world safety assessment of burosumab: a pharmacovigilance study utilizing the FDA adverse event reporting system. Orphanet Journal Rare Diseases. 21, 131. 10.1186/s13023-026-04267-9 41761237 PMC13059399

[B12] Chen Z. Y. Du Y. Guo H. (2026). Real-world safety of apremilast: a pharmacovigilance analysis using the FDA adverse event reporting system (FAERS). J. Am. Acad. Dermatology 94 (1), 330–332. 10.1016/j.jaad.2025.09.039 40962185

[B13] Das P. Ojha B. Das A. Pathak B. K. Mukhopadhyay K. (2025). Safety and efficacy of givinostat for patients with muscular dystrophy: a systematic review. Pharmacology 110, 1–13. 10.1159/000547936 40815117 PMC12503821

[B14] Duan D. Goemans N. Takeda S. Mercuri E. Aartsma-Rus A. (2021). Duchenne muscular dystrophy. Nat. Reviews. Dis. Primers 7 (1), 13. 10.1038/s41572-021-00248-3 33602943 PMC10557455

[B15] Gao H. Cao L. Liu C. (2025). Analysis and mining of dupilumab adverse events based on FAERS database. Sci. Reports 15 (1), 8597. 10.1038/s41598-025-92330-z 40074775 PMC11903887

[B16] He C. Z. Qiu Q. Lu S. J. Xue F. L. Liu J. Q. He Y. (2025). Adverse event reporting of faricimab: a disproportionality analysis of FDA adverse event reporting system (FAERS) database. Front. Pharmacology 16, 1521358. 10.3389/fphar.2025.1521358 40144657 PMC11936923

[B17] Hoffman K. B. Dimbil M. Erdman C. B. Tatonetti N. P. Overstreet B. M. (2014). The weber effect and the United States food and drug Administration's adverse event reporting system (FAERS): analysis of sixty-two drugs approved from 2006 to 2010. Drug Safety 37 (4), 283–294. 10.1007/s40264-014-0150-2 24643967 PMC3975089

[B18] Hong P. Zhou Q. (2025). Evaluation and study of adverse reactions to imiglucerase based on the FAERS database. Orphanet Journal Rare Diseases 20 (1), 406. 10.1186/s13023-025-03934-7 40775785 PMC12329963

[B19] Jama A. Alshudukhi A. A. Burke S. Dong L. Kamau J. K. Morris B. (2024). Exploring lipin1 as a promising therapeutic target for the treatment of Duchenne muscular dystrophy. J. Translational Medicine 22 (1), 664. 10.1186/s12967-024-05494-z 39014470 PMC11253568

[B20] Joshi A. Gawey L. Saadi C. Naidoo P. Rick J. W. Park S. Y. (2025). Postmarketing safety surveillance of adalimumab, secukinumab, and infliximab in hidradenitis suppurativa: an analysis of the FDA adverse events reporting system (FAERS) database. J. Am. Acad. Dermatology 92 (6), 1434–1435. 10.1016/j.jaad.2025.02.031 39978679

[B21] King W. M. Ruttencutter R. Nagaraja H. N. Matkovic V. Landoll J. Hoyle C. (2007). Orthopedic outcomes of long-term daily corticosteroid treatment in Duchenne muscular dystrophy. Neurology 68 (19), 1607–1613. 10.1212/01.wnl.0000260974.41514.83 17485648

[B22] Lamb Y. N. (2024). Givinostat: first approval. Drugs 84 (7), 849–856. 10.1007/s40265-024-02052-1 38967716

[B23] Le D. V. Tran H. Wetter D. A. Nguyen G. H. (2026). Drug-associated paronychia: pharmacovigilance signals from the U.S. food and drug administration adverse event reporting system (FAERS) and the canada vigilance adverse reaction database (CVARD). J. Am. Acad. Dermatology. 95, 208–211. 10.1016/j.jaad.2026.02.046 41713582

[B24] Li X. Zhang S. Q. Wang K. R. Wu S. N. Wang M. Y. Chen C. T. (2025). Chloroquine and hydroxychloroquine-related ocular adverse events in SLE treatment: a real-world disproportionality analysis based on FDA adverse event reporting system (FAERS). Front. Pharmacology 16, 1498814. 10.3389/fphar.2025.1498814 40661080 PMC12256490

[B25] Lin J. Cheng Y. Chen L. Chen M. Shen Z. (2025a). A real-world analysis of adverse event signals of cognitive and communication disorder in patients treated with janus kinase inhibitors based on the FAERS database. J. Am. Acad. Dermatology 93 (6), 1562–1564. 10.1016/j.jaad.2025.08.003 40783117

[B26] Lin Z. Yu X. Yang M. Xu J. Zhong J. (2025b). A real-world pharmacovigilance study of FDA adverse event reporting system (FAERS) events for bimekizumab. Front. Pharmacology 16, 1714173. 10.3389/fphar.2025.1714173 41560729 PMC12812977

[B27] Lu Z. Huang M. Liu L. Lin C. Lin W. Tan H. (2026). Drug-induced perioperative malignant hyperthermia: a real-world study based on the food and drug administration adverse event reporting system (FAERS) database. Int. Journal Surgery Lond. Engl. 112 (2), 2683–2695. 10.1097/js9.0000000000003609 41731858

[B28] Markati T. Oskoui M. Farrar M. A. Duong T. Goemans N. Servais L. (2022). Emerging therapies for Duchenne muscular dystrophy. Lancet. Neurology 21 (9), 814–829. 10.1016/s1474-4422(22)00125-9 35850122

[B29] McDonald C. M. Henricson E. K. Abresch R. T. Duong T. Joyce N. C. Hu F. (2018). Long-term effects of glucocorticoids on function, quality of life, and survival in patients with Duchenne muscular dystrophy: a prospective cohort study. Lancet 391 (10119), 451–461. 10.1016/s0140-6736(17)32160-8 29174484

[B30] Mendell J. R. Muntoni F. McDonald C. M. Mercuri E. M. Ciafaloni E. Komaki H. (2025). AAV gene therapy for Duchenne muscular dystrophy: the EMBARK phase 3 randomized trial. Nat. Medicine 31 (1), 332–341. 10.1038/s41591-024-03304-z 39385046 PMC11750718

[B31] Mercuri E. Vilchez J. J. Boespflug-Tanguy O. Zaidman C. M. Mah J. K. Goemans N. (2024). Safety and efficacy of givinostat in boys with Duchenne muscular dystrophy (EPIDYS): a multicentre, randomised, double-blind, placebo-controlled, phase 3 trial. Lancet. Neurology 23 (4), 393–403. 10.1016/s1474-4422(24)00036-x 38508835

[B32] Michel C. Scosyrev E. Petrin M. Schmouder R. (2017). Can disproportionality analysis of post-marketing case reports be used for comparison of drug safety profiles. Clin. Drug Investigation 37 (5), 415–422. 10.1007/s40261-017-0503-6 28224371

[B33] O'Connor O. A. Horwitz S. Masszi T. Van Hoof A. Brown P. Doorduijn J. (2015). Belinostat in patients with relapsed or refractory peripheral T-Cell lymphoma: results of the pivotal phase II BELIEF (CLN-19) study. J. Clinical Oncology Official Journal Am. Soc. Clin. Oncol. 33 (23), 2492–2499. 10.1200/JCO.2014.59.2782 PMC5087312 26101246

[B34] Pirih F. Lu J. Ye F. Bezouglaia O. Atti E. Ascenzi M. G. (2012). Adverse effects of hyperlipidemia on bone regeneration and strength. J. Bone Mineral Research The Official Journal Am. Soc. Bone Mineral Res. 27 (2), 309–318. 10.1002/jbmr.541 21987408 PMC3274629

[B35] Qu H. Li W. Wu Z. Wang Y. Feng T. Li N. (2024). Differences in hypersensitivity reactions and gadolinium deposition disease/symptoms associated with gadolinium exposure to gadolinium-based contrast agents: new insights based on global databases VigiBase, FAERS, and IQVIA-MIDAS. BMC Medicine 22 (1), 329. 10.1186/s12916-024-03537-2 39135199 PMC11321222

[B36] Rai S. Kim W. S. Ando K. Choi I. Izutsu K. Tsukamoto N. (2023). Oral HDAC inhibitor tucidinostat in patients with relapsed or refractory peripheral T-cell lymphoma: phase IIb results. Haematologica 108 (3), 811–821. 10.3324/haematol.2022.280996 36200417 PMC9973490

[B37] Ricotti V. Mandy W. P. Scoto M. Pane M. Deconinck N. Messina S. (2016). Neurodevelopmental, emotional, and behavioural problems in Duchenne muscular dystrophy in relation to underlying dystrophin gene mutations. Dev. Medicine Child Neurology 58 (1), 77–84. 10.1111/dmcn.12922 26365034

[B38] Tian L. Yu X. (2015). Lipid metabolism disorders and bone dysfunction--interrelated and mutually regulated (review). Mol. Medicine Reports 12 (1), 783–794. 10.3892/mmr.2015.3472 25760577 PMC4438959

[B39] Waite A. Brown S. C. Blake D. J. (2012). The dystrophin-glycoprotein complex in brain development and disease. Trends Neurosciences 35 (8), 487–496. 10.1016/j.tins.2012.04.004 22626542

[B40] Ye G. Ding L. Zhou Q. (2025). Remimazolam's clinical application and safety: a signal detection analysis based on FAERS data and literature support. PloS One 20 (8), e0330769. 10.1371/journal.pone.0330769 40845004 PMC12373250

[B41] Yin Y. Shu Y. Zhu J. Li F. Li J. (2022). A real-world pharmacovigilance study of FDA adverse event reporting system (FAERS) events for osimertinib. Sci. Reports 12 (1), 19555. 10.1038/s41598-022-23834-1 36380085 PMC9664039

[B42] Zhan H. Liu X. Piao S. Rong X. Guo J. (2023). Association between triglyceride-glucose index and bone mineral density in US adults: a cross sectional study. J. Orthopaedic Surgery Research 18 (1), 810. 10.1186/s13018-023-04275-6 37904197 PMC10614394

[B43] Zhou J. Zheng Y. Xu B. Long S. Zhu L. e. Liu Y. (2024). Exploration of the potential association between GLP-1 receptor agonists and suicidal or self-injurious behaviors: a pharmacovigilance study based on the FDA adverse event reporting system database. BMC Medicine 22 (1), 65. 10.1186/s12916-024-03274-6 38355513 PMC10865629

[B44] Zhu Z. Li Y. Zhu C. Dong Q. Zhang Y. Liu Z. (2025). Disproportionality analysis of interstitial lung disease associated with novel antineoplastic agents during breast cancer treatment: a pharmacovigilance study. EClinicalMedicine 82, 103160. 10.1016/j.eclinm.2025.103160 40166653 PMC11957809

