# Supplementary material for: Real-world safety profile of givinostat: an early post-marketing pharmacovigilance study based on the FAERS database
Source: Front Pharmacol. 2026 Jul 9;17:1861893. doi: 10.3389/fphar.2026.1861893 (PMC13392257; doi:10.3389/fphar.2026.1861893)
Supplement: Supplementary file 1 [file Table1.docx]

**Supplementary Table S1. Sensitivity analysis of givinostat-associated AE signals (retaining only PTs with ≥ 4 cases).**

| **SOC** | **PTs** | **Case Reports** | **ROR (95% CI)** | **PRR (95% CI)** | **χ2** | **IC (IC025)** | **EBGM (EBGM05)** |
| --- | --- | --- | --- | --- | --- | --- | --- |
| Investigations | Platelet Count Decreased | 97 | 61.58 (48.98–77.43) | 46.96 (39.46–55.90) | 4357.25 | 5.24 (4.95) | 46.66 (38.24) |
| Gastrointestinal disorders | Diarrhoea | 59 | 4.85 (3.68–6.40) | 4.29 (3.39–5.43) | 153.84 | 2.06 (1.69) | 4.28 (3.32) |
| Investigations | Blood Triglycerides Increased | 45 | 201.97 (147.61–276.34) | 179.47 (135.74–237.29) | 7792.96 | 5.91 (5.49) | 175.04 (130.69) |
| Gastrointestinal disorders | Nausea | 31 | 2.23 (1.54–3.21) | 2.13 (1.52–2.99) | 19.32 | 1.07 (0.56) | 2.13 (1.50) |
| Gastrointestinal disorders | Vomiting | 30 | 3.46 (2.39–5.02) | 3.28 (2.32–4.63) | 48.62 | 1.66 (1.15) | 3.28 (2.29) |
| Gastrointestinal disorders | Abdominal Discomfort | 28 | 8.49 (5.78–12.47) | 7.97 (5.57–11.39) | 171.97 | 2.83 (2.30) | 7.96 (5.50) |
| Blood and lymphatic system disorders | Thrombocytopenia | 28 | 13.28 (9.04–19.51) | 12.43 (8.69–17.77) | 295.32 | 3.37 (2.84) | 12.41 (8.57) |
| Gastrointestinal disorders | Abdominal Pain Upper | 20 | 5.11 (3.26–8.01) | 4.90 (3.20–7.52) | 62.71 | 2.16 (1.54) | 4.90 (3.16) |
| Investigations | Weight Increased | 20 | 4.51 (2.88–7.08) | 4.34 (2.83–6.65) | 51.96 | 2.00 (1.38) | 4.34 (2.80) |
| General disorders and administration site conditions | Pyrexia | 16 | 2.46 (1.49–4.06) | 2.40 (1.49–3.88) | 13.30 | 1.20 (0.51) | 2.40 (1.47) |
| Gastrointestinal disorders | Gastrointestinal Disorder | 15 | 5.59 (3.34–9.36) | 5.42 (3.30–8.90) | 54.37 | 2.24 (1.53) | 5.41 (3.26) |
| Injury, poisoning and procedural complications | Contusion | 13 | 6.84 (3.93–11.89) | 6.65 (3.89–11.35) | 62.65 | 2.46 (1.69) | 6.64 (3.86) |
| Musculoskeletal and connective tissue disorders | Myalgia | 13 | 4.43 (2.55–7.69) | 4.31 (2.53–7.37) | 33.33 | 1.94 (1.17) | 4.31 (2.50) |
| Gastrointestinal disorders | Abdominal Pain | 11 | 2.46 (1.35–4.48) | 2.42 (1.35–4.34) | 9.29 | 1.19 (0.36) | 2.42 (1.34) |
| Skin and subcutaneous tissue disorders | Alopecia | 10 | 3.08 (1.64–5.77) | 3.03 (1.64–5.58) | 13.68 | 1.46 (0.59) | 3.03 (1.63) |
| Respiratory, thoracic and mediastinal disorders | Epistaxis | 7 | 5.60 (2.65–11.83) | 5.52 (2.65–11.51) | 25.97 | 2.08 (1.05) | 5.52 (2.63) |
| Injury, poisoning and procedural complications | Lower Limb Fracture | 5 | 9.98 (4.13–24.13) | 9.87 (4.13–23.60) | 39.85 | 2.45 (1.24) | 9.86 (4.10) |
| Infections and infestations | Viral Infection | 5 | 6.05 (2.50–14.62) | 5.99 (2.50–14.31) | 20.79 | 2.04 (0.84) | 5.98 (2.49) |
| Psychiatric disorders | Anger | 4 | 9.51 (3.55–25.47) | 9.42 (3.55–25.00) | 30.11 | 2.28 (0.95) | 9.41 (3.53) |
| Investigations | Blood Cholesterol Increased | 4 | 3.92 (1.46–10.50) | 3.89 (1.47–10.32) | 8.61 | 1.56 (0.23) | 3.89 (1.46) |
| General disorders and administration site conditions | Crying | 4 | 8.97 (3.35–24.03) | 8.89 (3.35–23.59) | 28.01 | 2.24 (0.91) | 8.88 (3.33) |
| Psychiatric disorders | Emotional Disorder | 4 | 7.16 (2.67–19.19) | 7.10 (2.68–18.84) | 20.99 | 2.08 (0.75) | 7.10 (2.66) |
| Injury, poisoning and procedural complications | Femur Fracture | 4 | 10.46 (3.90–28.03) | 10.37 (3.91–27.50) | 33.83 | 2.34 (1.01) | 10.35 (3.89) |
| Infections and infestations | Gastroenteritis Viral | 4 | 8.57 (3.20–22.96) | 8.50 (3.20–22.54) | 26.45 | 2.21 (0.88) | 8.49 (3.19) |
| Metabolism and nutrition disorders | Increased Appetite | 4 | 8.07 (3.01–21.63) | 8.00 (3.02–21.23) | 24.52 | 2.17 (0.84) | 8.00 (3.00) |
| Skin and subcutaneous tissue disorders | Petechiae | 4 | 24.46 (9.12–65.60) | 24.22 (9.12–64.34) | 88.79 | 2.76 (1.42) | 24.14 (9.06) |
