# Supplementary material for: Real-world safety profile of givinostat: an early post-marketing pharmacovigilance study based on the FAERS database
Source: Front Pharmacol. 2026 Jul 9;17:1861893. doi: 10.3389/fphar.2026.1861893 (PMC13392257; doi:10.3389/fphar.2026.1861893)
Supplement: Supplementary file 2 [file Table2.docx]

**Supplementary Table S2.** Exploratory signals excluded from the primary analysis due to low report counts (N ≤ 3).

| **SOC** | **PTs** | **Case Reports** | **ROR (95% CI)** | **PRR (95% CI)** | **χ2** | **IC (IC025)** | **EBGM (EBGM05)** |
| --- | --- | --- | --- | --- | --- | --- | --- |
| Blood and lymphatic system disorders | Anaemia Macrocytic | 3 | 77.69 (24.80–243.40) | 77.12 (24.82–239.58) | 222.97 | 2.70 (1.19) | 76.29 (24.61) |
| Psychiatric disorders | Frustration Tolerance Decreased | 3 | 14.60 (4.68–45.53) | 14.50 (4.69–44.83) | 37.66 | 2.31 (0.80) | 14.47 (4.67) |
| Investigations | Laboratory Test Abnormal | 3 | 5.42 (1.74–16.87) | 5.38 (1.74–16.63) | 10.71 | 1.73 (0.22) | 5.38 (1.74) |
| Psychiatric disorders | Mood Swings | 3 | 7.32 (2.35–22.82) | 7.28 (2.36–22.48) | 16.24 | 1.94 (0.43) | 7.27 (2.34) |
| Psychiatric disorders | Panic Attack | 3 | 4.73 (1.52–14.73) | 4.70 (1.52–14.52) | 8.75 | 1.62 (0.11) | 4.70 (1.52) |
