# Supplementary material for: Real-world safety profile of givinostat: an early post-marketing pharmacovigilance study based on the FAERS database
Source: Front Pharmacol. 2026 Jul 9;17:1861893. doi: 10.3389/fphar.2026.1861893 (PMC13392257; doi:10.3389/fphar.2026.1861893)
Supplement: Supplementary file 3 [file Table5.docx]

**Supplementary Table S5.** Time-to-onset (TTO) analysis of early major adverse events associated with givinostat.

| Adverse Event Category | Valid Cases (N) | Median TTO (days) | IQR (days) | Range (days) |
| --- | --- | --- | --- | --- |
| Gastrointestinal disorders | 20 | 2.0 | 0.0 - 15.2 | 0 - 105 |
| Hematological toxicity | 19 | 19.0 | 14.0 - 39.5 | 1 - 69 |
| Hypertriglyceridemia | 12 | 21.5 | 14.0 - 52.0 | 0 - 144 |

TTO, time-to-onset; IQR, interquartile range. Note: The number of valid cases (N) for TTO calculation is smaller than the total reported cases (e.g., Table 6) because only reports containing both complete therapy start dates and adverse event onset dates were included in this temporal analysis.
