# Supplementary material for: Real-world safety profile of givinostat: an early post-marketing pharmacovigilance study based on the FAERS database
Source: Front Pharmacol. 2026 Jul 9;17:1861893. doi: 10.3389/fphar.2026.1861893 (PMC13392257; doi:10.3389/fphar.2026.1861893)
Supplement: Supplementary file 4 [file Table3.docx]

**Supplementary Table S3.** Year-stratified distribution of significant givinostat-associated adverse event signals.

| **SOC** | **PTs** | **Total** | **Cases in 2024** | **Cases in 2025** | **Unknown** |
| --- | --- | --- | --- | --- | --- |
| Investigations | Platelet Count Decreased | 97 | 8 | 82 | 7 |
| Gastrointestinal disorders | Diarrhoea | 59 | 2 | 52 | 5 |
| Investigations | Blood Triglycerides Increased | 45 | 0 | 42 | 3 |
| Gastrointestinal disorders | Nausea | 31 | 6 | 19 | 6 |
| Gastrointestinal disorders | Vomiting | 30 | 3 | 20 | 7 |
| Gastrointestinal disorders | Abdominal Discomfort | 28 | 4 | 22 | 2 |
| Blood and lymphatic system disorders | Thrombocytopenia | 28 | 2 | 24 | 2 |
| Gastrointestinal disorders | Abdominal Pain Upper | 20 | 0 | 13 | 7 |
| Investigations | Weight Increased | 20 | 0 | 1 | 19 |
| General disorders and administration site conditions | Pyrexia | 16 | 0 | 9 | 7 |
| Gastrointestinal disorders | Gastrointestinal Disorder | 15 | 1 | 14 | 0 |
| Injury, poisoning and procedural complications | Contusion | 13 | 0 | 13 | 0 |
| Musculoskeletal and connective tissue disorders | Myalgia | 13 | 0 | 10 | 3 |
| Gastrointestinal disorders | Abdominal Pain | 11 | 0 | 10 | 1 |
| Skin and subcutaneous tissue disorders | Alopecia | 10 | 0 | 10 | 0 |
| Respiratory, thoracic and mediastinal disorders | Epistaxis | 7 | 0 | 7 | 0 |
| Injury, poisoning and procedural complications | Lower Limb Fracture | 5 | 0 | 2 | 3 |
| Infections and infestations | Viral Infection | 5 | 0 | 3 | 2 |
| Psychiatric disorders | Anger | 4 | 0 | 0 | 4 |
| Investigations | Blood Cholesterol Increased | 4 | 0 | 2 | 2 |
| General disorders and administration site conditions | Crying | 4 | 1 | 0 | 3 |
| Psychiatric disorders | Emotional Disorder | 4 | 1 | 0 | 3 |
| Injury, poisoning and procedural complications | Femur Fracture | 4 | 0 | 2 | 2 |
| Infections and infestations | Gastroenteritis Viral | 4 | 0 | 2 | 2 |
| Metabolism and nutrition disorders | Increased Appetite | 4 | 0 | 0 | 4 |
| Skin and subcutaneous tissue disorders | Petechiae | 4 | 0 | 4 | 0 |
| Blood and lymphatic system disorders | Anaemia Macrocytic | 3 | 0 | 3 | 0 |
| Psychiatric disorders | Frustration Tolerance Decreased | 3 | 0 | 0 | 3 |
| Investigations | Laboratory Test Abnormal | 3 | 0 | 3 | 0 |
| Psychiatric disorders | Mood Swings | 3 | 0 | 0 | 3 |
| Psychiatric disorders | Panic Attack | 3 | 0 | 1 | 2 |
