# Supplementary material for: Real-world safety profile of givinostat: an early post-marketing pharmacovigilance study based on the FAERS database
Source: Front Pharmacol. 2026 Jul 9;17:1861893. doi: 10.3389/fphar.2026.1861893 (PMC13392257; doi:10.3389/fphar.2026.1861893)
Supplement: Supplementary file 5 [file Table4.docx]

****Supplementary Table S4.** Feasibility assessment for active-comparator analysis: FAERS report counts and methodological limitations of potential comparator drugs.**

| Drug Category | Comparator Drug | Total FAERS Reports | Feasibility Limitation for Active-Comparator Analysis |
| --- | --- | --- | --- |
| DMD-indicated agents | Deflazacort | 4,021 | Sparse overlap; high variance in background exposure resulting in unstable estimates for novel givinostat signals. |
|  | Eteplirsen | 2,115 | Unstable estimates (N < 3) / wide 95% CI; zero-cell problems for specific target AEs. |
|  | Golodirsen | 415 | Zero-cell / Sparse Data: Total report count is critically low, rendering statistical estimates impossible. |
| Other HDAC inhibitors | Vorinostat | 2,285 | Zero-cell problem in 2x2 contingency table for specific target AEs. |
|  | Romidepsin | 1,578 | Sparse Data / Indication Confounding: Specific oncology background heavily confounds comparative safety profiling against pediatric DMD. |
|  | Panobinostat | 621 | Zero-cell / Sparse Data: Extremely low total report count; severe baseline demographic and disease-state mismatch. |
| Standard of care (Corticosteroids) | Prednisone | 188,312 | Massive Background Confounding: Huge report volume across diverse systemic indications obscures DMD-specific baseline risks (high risk of Simpson's Paradox). |
